# Application of thrombelastography (TEG) for safety evaluation of tranexamic acid in primary total joint arthroplasty

**DOI:** 10.1186/s13018-019-1250-6

**Published:** 2019-07-15

**Authors:** Xiang-Dong Wu, Yu Chen, Mian Tian, Yao He, Yu-Zhang Tao, Wei Xu, Qiang Cheng, Cheng Chen, Wei Liu, Wei Huang

**Affiliations:** 1 Department of Orthopaedic Surgery, The First Affiliated Hospital of Chongqing Medical University, No. 1, Youyi Road, Yuanjiagang, Yuzhong District, Chongqing, 400016 China; 2 Department of Orthopedic Surgery, Peking Union Medical College Hospital, Chinese Academy of Medical Sciences & Peking Union Medical College, Beijing, 100730 China; 3 Department of Orthopaedic Surgery, Dianjiang People’s Hospital, Chongqing, 400060 China; 4 Department of Orthopaedic Surgery, Banan People’s Hospital of Chongqing, Chongqing, 400320 China

**Keywords:** Multiple-dose, Safety, Thromboelastography, Total hip arthroplasty, Total knee arthroplasty, Tranexamic acid

## Abstract

**Background:**

Questions remain, mainly concerning whether tranexamic acid (TXA) is truly safe since all available trials were underpowered to identify clinically important differences. The objective of this study is to evaluate the safety of TXA by using a novel technique—thromboelastography (TEG).

**Methods:**

A retrospective review was conducted on 359 consecutive patients who underwent primary total hip arthroplasty (THA) or total knee arthroplasty (TKA) and received multiple-dose or single-dose of TXA at a tertiary academic center. TEG parameters, TEG coagulation status, conventional coagulation test parameters, and incidence of thrombotic events were used for safety evaluation.

**Results:**

Compared with single-dose cohort, patients who received multiple-dose of TXA had consistent statistically significant shortened R times on post-operative day 1 (POD1) and POD3 in both THA (POD1: 4.06 ± 0.71 s versus 4.45 ± 1.28 s, *P* = 0.011; POD3: 4.36 ± 0.83 s versus 5.12 ± 1.64 s, *P* < 0.0001) and TKA (POD1: 3.90 ± 0.73 s versus 4.29 ± 0.92 s, *P* = 0.011; POD3: 4.24 ± 0.94 s versus 4.65 ± 1.07 s, *P* = 0.023), while the K, α-angle, and MA values were similar during the perioperative period. TEG coagulation status analysis indicated that patients were significantly (*P* = 0.003) more likely with hypercoagulable status during the course of multiple-dose TXA. Conventional coagulation test parameters were similar. Only one patient developed calf vein thrombosis in the multiple-dose cohort.

**Conclusions:**

Multiple-dose of TXA was associated with aggravated hypercoagulable state when compared with single-dose of TXA, but this prothrombotic state does not provoke thrombosis when combined with appropriate anticoagulant therapy. Therefore, multiple-dose of TXA remains safe and could be recommended for clinical practice. Potential benefits and possible risks should be trade-off when considering increasing the dosage and frequency of TXA on the present basis.

**Trial registration:**

ChiCTR1800015422.

## Introduction

Despite more than two decades of experience with tranexamic acid (TXA) use, and reports from dozens of clinical trials enrolling thousands of patients to examine the efficacy as well as safety of application of TXA in arthroplasty, still there remain some critical questions [1, 2]. Previous studies have proposed various administration regimens of TXA, and proved its efficacy in reducing blood loss, post-operative drainage volume, transfusion rate, inflammatory response, etc., and suppose it is safe with no significantly increased incidence of venous thromboembolic (VTE) events [1, 3–11]. However, a longstanding issue of concern is whether application of TXA will promote a hypercoagulable state and increase the risk of VTE. A recent published meta-analysis evaluated and established a basis for the safety recommendation of TXA in clinical practice guidelines [12]. Nevertheless, since the rarity of thromboembolic events and similar incidence of VTE in patients treated with or without TXA, the aggregated large number of level-I evidence studies remain lacking in sufficient statistical power to detect the clinically important differences and reach a firm conclusion [12–14]. To overcome this issue, safety evaluation of TXA from completely different perspectives should be considered [13, 14].

Viscoelastic hemostatic assays (VHA) technology, such as thromboelastography (TEG) and thromboelastometry (ROTEM), are whole blood tests that depict functional coagulation both numerically and graphically [15, 16]. Thus, VHA could be used to monitor the changes of blood coagulability after use of TXA in patients undergoing elective arthroplasty.

Therefore, we conducted a retrospective cohort study to assess the safety of TXA by comparing multiple-dose versus single-dose of TXA in patients undergoing primary total hip arthroplasty (THA) and total knee arthroplasty (TKA). We hypothesized that multiple-dose of TXA will not increase the incidence of VTE events, but will cause severe hypercoagulability and increased risk of VTE.

## Materials and methods

### Study design and patients

The ethic approval of this study was obtained from the Institutional Review Board. The research as part of our registered project on Chinese Clinical Trial Registry (ChiCTR1800015422) is being reported in line with Strengthening the Reporting Of Cohort Studies in Surgery (STROCSS) [17].

We performed a retrospective review of consecutive patients who received THA or TKA at a single tertiary academic center between September 2014 and December 2017. Inclusion criteria consisted of adult patients who underwent elective unilateral primary THA or TKA, and received single-dose or multiple-dose of TXA as described below. Exclusion criteria included (i) patients with moderate or severe anemia (Hb < 9 g/dL); (ii) patients who underwent simultaneous bilateral arthroplasty, emergent debridement, or revision procedure; (iii) with severe pre-operative varus or valgus deformity and received complex osteotomies; (iv) with a documented history of thromboembolic events (including nonfatal myocardial infarction, pulmonary embolism, stroke, or bowel infarction); (v) pre-operative serious cardiac or respiratory disease; (vi) congenital or acquired thrombophilia or coagulopathy; (vii) severe renal impairment or liver insufficiency; and (viii) allergy to TXA and discontinued intravenous (IV) solution or did not receive TXA.

### TXA administration protocol

In the single-dose cohort, patients received a single bolus of 1.5 g IV TXA 30 min before incision. While in the multiple-dose cohort, patients received a bolus of 1.5 g IV TXA 30 min before incision, 1 g topical (intra-articular) TXA injected after capsule closure during surgery, and 1 g IV TXA administrated at 3 h, 12 h, 24 h, 48 h, and 72 h after surgery, respectively.

### Surgical procedures

All procedures were performed by two senior orthopedic surgeons under general anesthesia. THA was performed through a standard posterolateral approach and no drainage tube was applied. TKA was performed through a standard medial parapatellar approach under a bloodless field provided by a pneumatic tourniquet. The tourniquet was applied throughout the whole course and was not released until skin closure. A vacuum drainage tube was routinely in place for 24 h, and removal depends on the amount of drainage.

### Perioperative management

A restrictive transfusion strategy (Hb < 7.0 g/dL or symptomatic anemia with a Hb ≥ 7.0 g/dL) was applied for allogenic red blood cell transfusion [18]. A combined mechanical and pharmacological prophylaxis was adopted to potentiate the overall efficacy of VTE prevention. Most of the patients were taking 10 mg rivaroxaban orally once a day, while some patients received low molecular weight heparin during hospitalization, and bridging to rivaroxaban after discharge. Routine Doppler ultrasound screening for deep vein thrombosis (DVT) was conducted at the time of discharge, and contrast-enhanced chest computed tomography scan would be performed only when pulmonary embolism (PE) was strongly suspected based on clinical symptoms.

### Outcome measurements

Safety outcomes included TEG parameters, TEG coagulation status analysis, traditional coagulation test parameters (prothrombin time (PT), activated partial thromboplastin time (APTT), thromboplastin time (TT), fibrinogen (Fbg) concentration), and incidence of DVT. All blood tests were routinely performed pre-operatively (Pre), post-operative day 1 (POD1), POD3, POD5, and POD7.

### Thromboelastography

Standard coagulation measures have limited value in arthroplasty as they reflect deficiencies in procoagulant factors, without balancing concurrent deficiencies of anticoagulant factors such as thrombomodulin [19]. Indeed, there has been a persistence of hypercoagulability state after arthroplasty, but conventional coagulation tests would display normal parameters and suggest a balanced coagulation in standard conditions [20, 21]. TEG is a global hemostasis assessment technique that could provide additional information on the hemostatic process and coagulability changes [22, 23], and recent studies have shown that TEG was an effective way to identify hypercoagulability or reflect the variation of coagulability [24–26]. Therefore, TEG could be used to detect the potential marginal changes of coagulability following the use of TXA.

TEG parameters mainly include reaction time (R), kinetics (K), alpha-angle (α-angle), maximum amplitude (MA), and coagulation index (CI) (Table 1). CI was dropped because it is a linear combination of R, K, α-angle, and MA values, which could appear normal but actually origin from mixed results, hyper- and hypo-coagulable of R or MA parameter abnormality. According to the manufacturer, the coagulation status could be simply classified into four types according to the parameters (Table 2). Standard citrated kaolin-activated TEGs were performed using TEG® Hemostasis Analyzer, Model 5000 (Haemonetics Corporation, Braintree, MA, USA).

**Table 1:** The summary of major thromboelastography parameters

Parameters	Abbreviation	Definition	Represent
Reaction time	R	Time until formation of critical mass of thrombin	Enzymatic reaction function
Kinetics	K	The speed of thrombus formation	Clot kinetics
Alpha-Angle	α-Angle	The rapidity of fibrin build-up and cross-linking	Fibrinogen level
Maximum amplitude	MA	Direct function of the maximum dynamic properties of fibrin and platelet bonding via GPIIb/IIIa	Maximum platelet function
Coagulation Index	CI	Global index of coagulation status	A linear combination of R, K, α-angle, and MA values

**Table 2:** The simplified classification of coagulation status by thromboelastography

Coagulation status	*R* (min)	MA (mm)
Normal	5–10	50–70
Factor hypercoagulability (enzymatic)	< 5	≤ 70
Factor (enzymatic) and platelet hypercoagulability	< 5	> 70
Platelet hypercoagulability	≥ 5	> 70

*R* reaction time, *MA* maximum amplitude

### Statistical analysis

All data were managed with Excel (Microsoft Corporation, WA, USA), and statistical analyses were performed with SPSS, version 21.0 software (SPSS Inc., Chicago, IL, USA). For continuous outcomes, Student *t* test was used to compare independent normally distributed numerical variables, and Wilcoxon Mann-Whitney *U* test was used for non-normal distribution or unequal variance. For dichotomous outcomes, Pearson chi-square test or Fisher’s exact test was used to compare the categorical variables. And *P* < 0.05 was considered as statistically significant.

## Results

### Patients characteristics

A total of 359 patients (193 hips, 166 knees) who underwent primary THA or TKA were eligible for the study. Patients were divided into four groups based on the type of surgery and dose regimens of TXA. Baseline clinical characteristics and demographics of the patients are presented in Table 3. There was no significant difference between the groups with respect to gender, BMI, ASA classification, or other perioperative data.

**Table 3:** Patient demographics for primary total hip and knee arthroplasty

THA	Multiple-dose TXA (*N* = 65)	Single-dose TXA (*N* = 128)	*P* value
Age (years)	65.52 ± 14.30	64.84 ± 14.16	0.754
Gender (male/female)	29/36	54/74	0.761
Height (m)	1.62 ± 0.69	1.60 ± 0.07	0.087
Weight (kg)	61.38 ± 9.36	58.74 ± 10.14	0.095
BMI (kg/m^2^)	23.49 ± 3.26	22.94 ± 3.62	0.327
Drink	20/65	29/128	0.294
Smoke	21/65	28/128	0.161
Diagnosis			
AVN	20	34	0.816
DDH	13	28	
FNF	20	46	
ITF	2	6	
OA	6	9	
RA	3	5	
FAI	1	0	
ASA grade	2.50 ± 0.72	2.38 ± 0.65	0.215
Operated side (left/right)	29/36	74/54	0.094
Operation time (min)	84.00 ± 29.32	80.34 ± 30.71	0.428
Length of hospital stay	14.63 ± 7.08	14.93 ± 9.01	0.816
Postoperative hospital stay	8.65 ± 6.49	9.27 ± 8.15	0.595
TKA	Multiple-dose TXA (*N* = 70)	Single-dose TXA (*N* = 96)	*P* value
Age (years)	68.33 ± 7.40	67.28 ± 8.53	0.411
Gender (male/female)	11/59	26/70	0.092
Height (m)	1.57 ± 0.06	1.58 ± 0.06	0.542
Weight (kg)	63.19 ± 9.44	60.97 ± 9.68	0.157
BMI (kg/m^2^)	25.66 ± 3.34	24.48 ± 3.92	0.057
Diagnosis			
OA	66	83	0.244
RA	4	11	
GA	0	2	
ASA grade	2.27 ± 0.48	2.17 ± 0.52	0.176
Operated side (left/right)	36/34	36/60	0.083
Operation time (min)	94.61 ± 28.07	95.54 ± 28.63	0.836
Length of hospital stay	14.87 ± 8.16	14.98 ± 6.60	0.925
Postoperative hospital stay	8.49 ± 7.40	8.94 ± 5.51	0.653

*ASA* American Society of Anesthesiologists physical status classification system, *AVN* avascular necrosis, *BMI* body mass index, *DDH* developmental dysplasia of the hip, *FAI* femoroacetabular impingement, *FNF* femoral neck fracture, *GA* gout arthritis, *ITF* intertrochanteric fracture, *OA* osteoarthritis, *RA* rheumatoid arthritis, *THA* total hip arthroplasty, *TKA* total knee arthroplasty, *TXA* tranexamic acid

### Safety outcomes

**Fig. 1:**
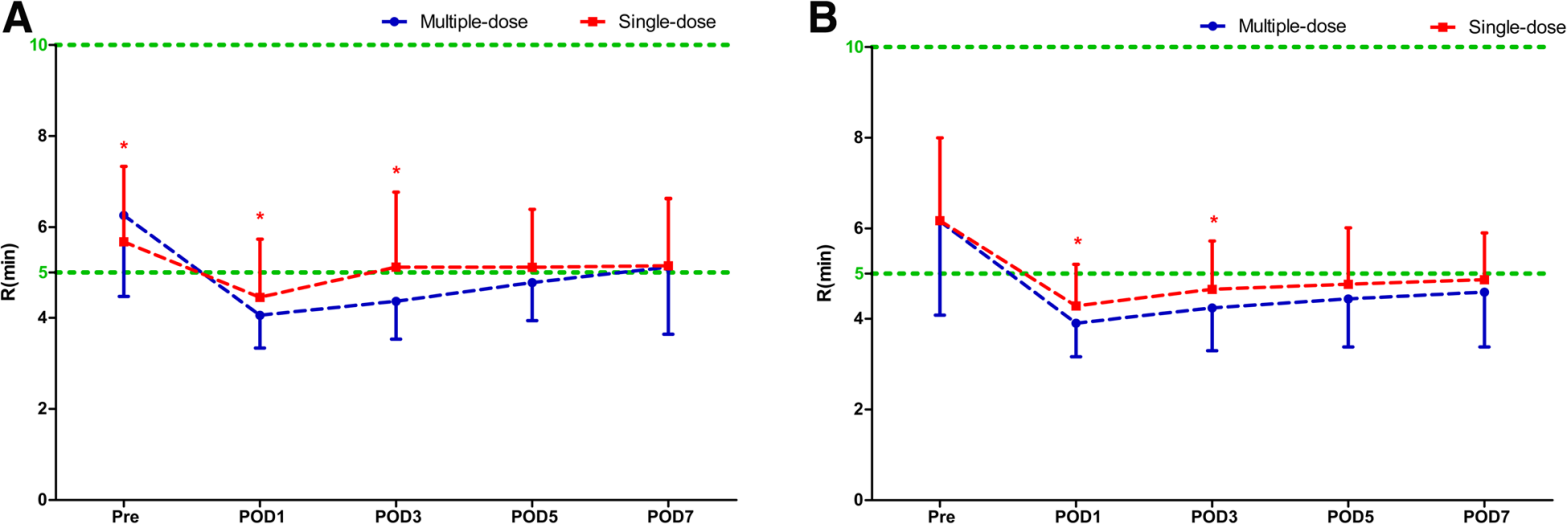
The perioperative *R* values of THA (**a**) and TKA (**b**). The asterisks indicate values that were significantly different between the multiple-dose and single-dose cohorts. Horizontal green dotted lines indicate the normal range (upper green line for upper limit, lower green line for lower limit). *R* reaction time, *THA* total hip arthroplasty, *TKA* total knee arthroplasty, *Pre* pre-operation, *POD1* post-operative day 1, *POD3* post-operative day 3, *POD5* post-operative day 5, *POD7* post-operative day 7

**Fig. 2:**
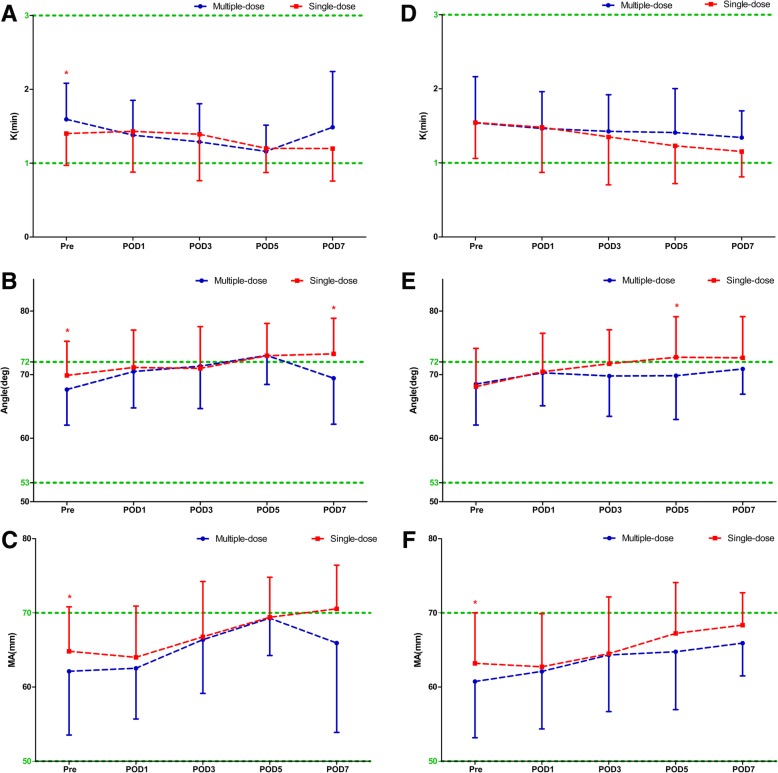
The perioperative TEG parameters (K, α-angle and MA values) of THA (**a**–**c**) and TKA (**d**–**f**). The asterisks indicate values that were significantly different between the multiple-dose and single-dose cohorts. Horizontal green dotted lines indicate the normal range (upper green line for upper limits, lower green line for lower limits). *K* kinetics, *MA* maximum amplitude, *TEG* thromboelastography, *THA* total hip arthroplasty, *TKA* total knee arthroplasty, *Pre* pre-operation, *POD1* post-operative day 1, *POD3* post-operative day 3, *POD5* post-operative day 5, *POD7* post-operative day 7

**Fig. 3:**
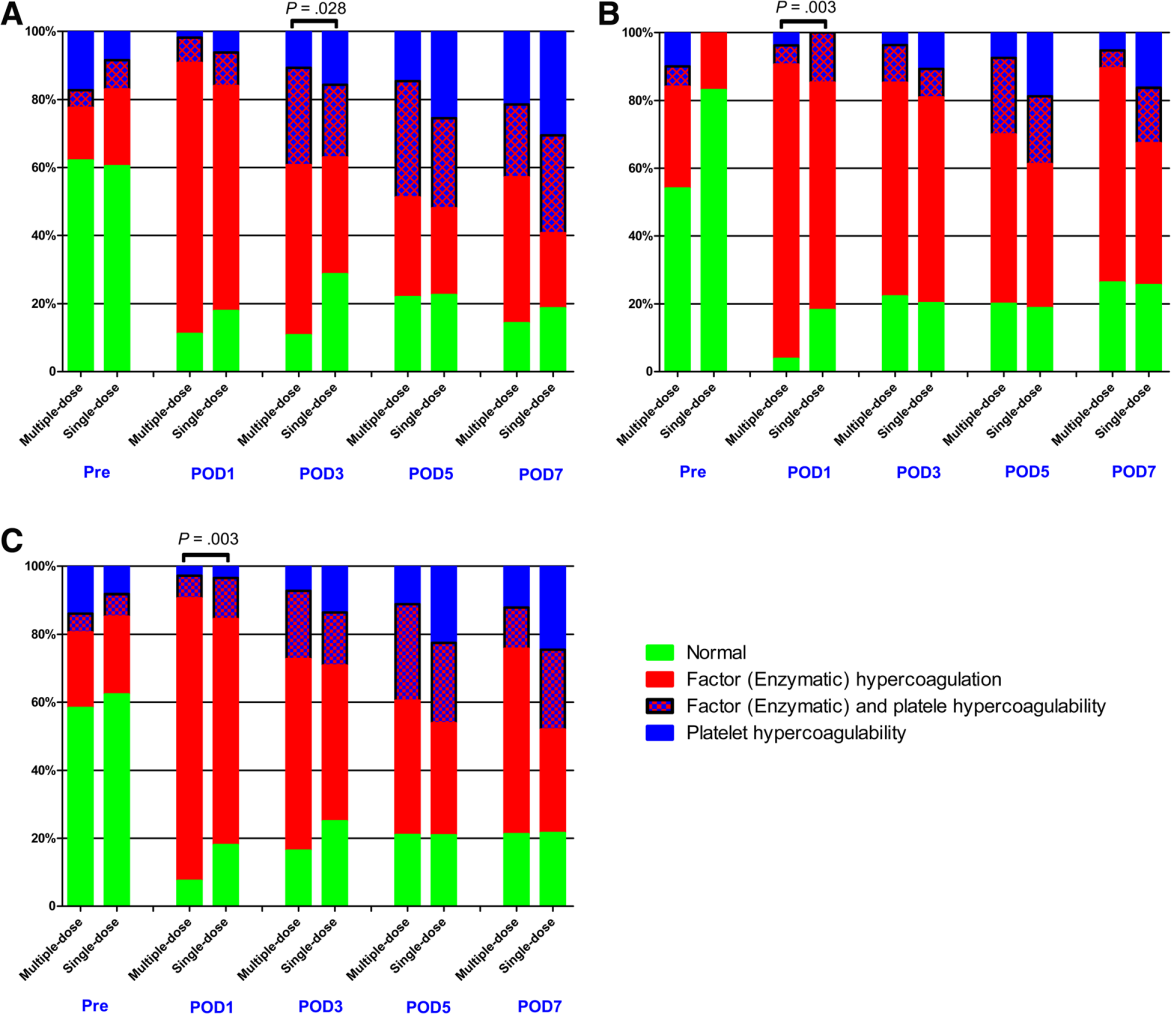
Proportion of different status of coagulability at different time points of THA (**a**), TKA (**b**), and TJA (**c**). *THA* total hip arthroplasty, *TKA* total knee arthroplasty, *TJA* total joint arthroplasty, *Pre* pre-operation, *POD1* post-operative day 1, *POD3* post-operative day 3, *POD5* post-operative day 5, *POD7* post-operative day 7

**Fig. 4:**
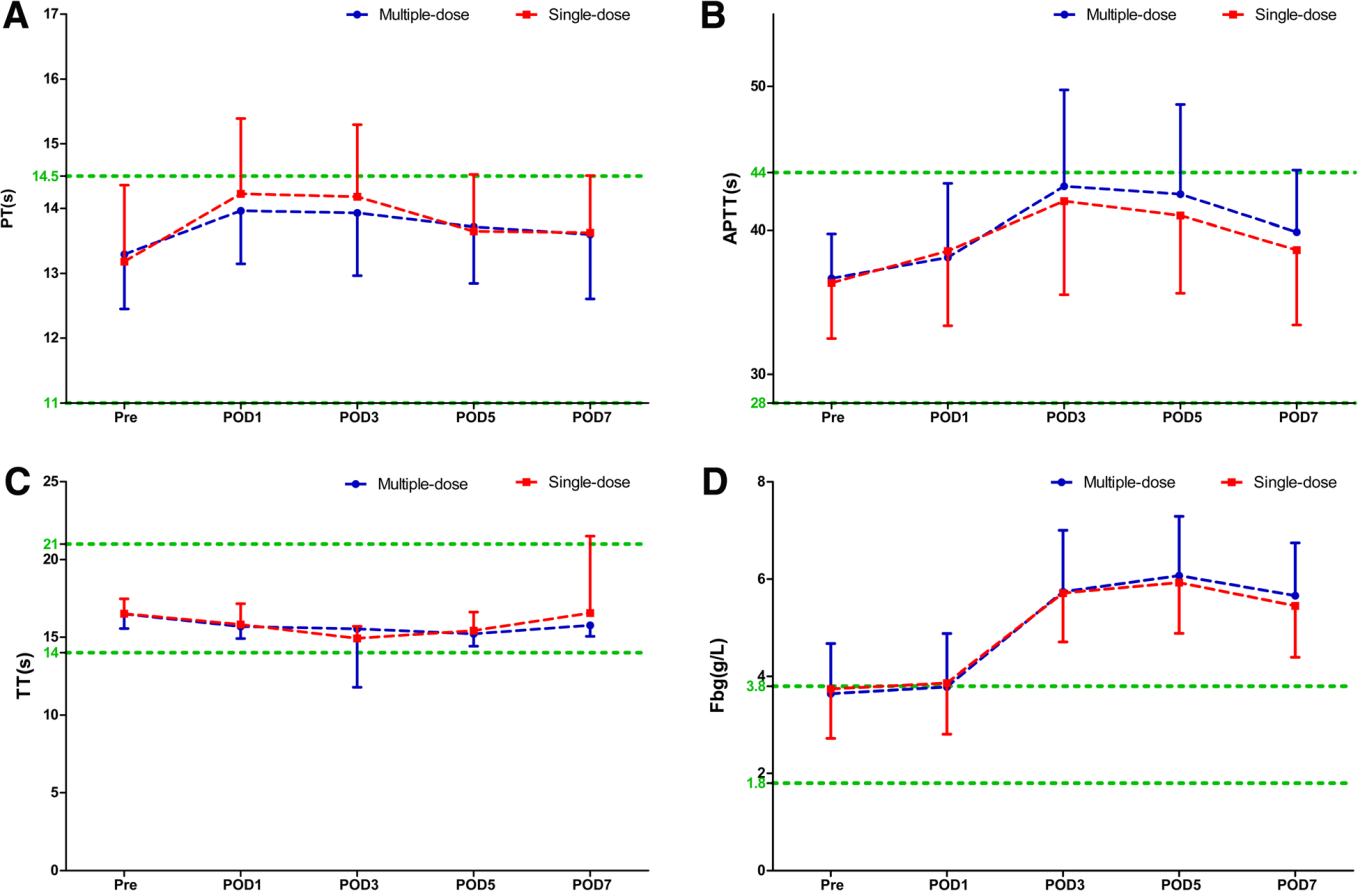
Conventional coagulation test parameters PT (**a**), APTT (**b**), TT (**c**), Fbg (**d**) of THA. The asterisks indicate values that were significantly different between the multiple-dose and single-dose cohorts. Horizontal green dotted lines indicate the normal range (upper green line for upper limit, lower green line for lower limit). *TT* thrombin time, *APTT* activated partial thromboplastin time, *PT* prothrombin time, *Fbg* fibrinogen, *THA* total hip arthroplasty, *Pre* pre-operation, *POD1* post-operative day 1, *POD3* post-operative day 3, *POD5* post-operative day 5, *POD7* post-operative day 7

**Fig. 5:**
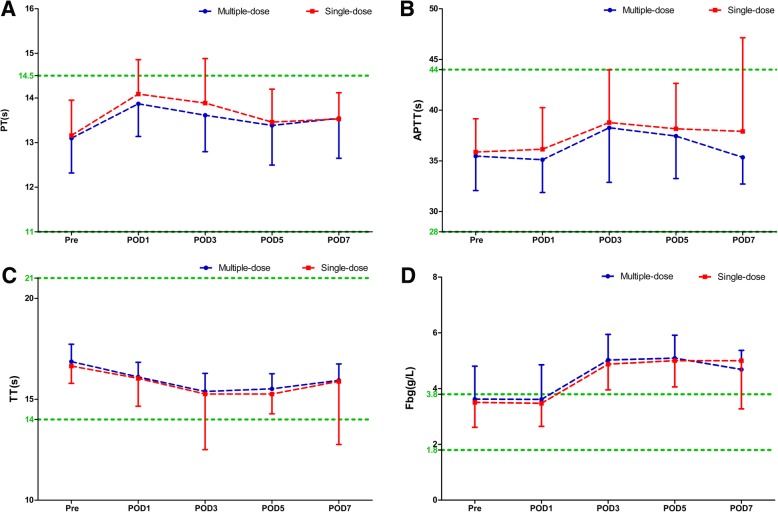
Conventional coagulation test parameters PT (**a**), APTT (**b**), TT (**c**), Fbg (**d**) of TKA. The asterisks indicate values that were significantly different between the multiple-dose and single-dose cohorts. Horizontal green dotted lines indicate the normal range (upper green line for upper limit, lower green line for lower limit). *TT* thrombin time, *APTT* activated partial thromboplastin time, *PT* prothrombin time, *Fbg* fibrinogen, *TKA* total knee arthroplasty, *Pre* pre-operation, *POD1* post-operative day 1, *POD3* post-operative day 3, *POD5* post-operative day 5, *POD7* post-operative day 7

Compared with the single-dose cohort, patients in the multiple-dose cohort had consistent statistically significant shortened R times on POD 1 and POD3 in both THA (POD1: 4.06 ± 0.71 s versus 4.45 ± 1.28 s, *P* = 0.011; POD3: 4.36 ± 0.83 s versus 5.12 ± 1.64 s, *P* < 0.0001) and TKA (POD1: 3.90 ± 0.73 s versus 4.29 ± 0.92 s, *P* = 0.011; POD3: 4.24 ± 0.94 s versus 4.65 ± 1.07 s, *P* = 0.023) (Fig. 1), while the K, α-angle, and MA values were similar during the perioperative period (Fig. 2). The distribution of different hypercoagulable states between the two dosing regimens of TXA in patient undergoing THA, TKA, and total joint arthroplasty (TJA) are shown in Fig. 3. Significant differences in the coagulation status categories between the two regimens of TXA were observed in THA patients (*P* = 0.028) on POD3, in TKA (*P* = 0.003) and TJA (*P* = 0.003) patients on POD1, which suggested that patients who received multiple-dose TXA were significantly more likely with hypercoagulable status. There was no significant difference of the conventional coagulation test parameters between the single-dose and multiple-dose cohorts (Figs. 4 and 5). DVT was detected in one patient who received THA in the multiple-dose cohort on POD12 by means of an ultrasound scan. This patient with sarcopenia experienced long-term bed confinement and reduced mobility in hospital. No episode of other adverse events such PE, stroke, or cardiac infraction occurred during hospitalization.

## Discussion

### Main findings

The main finding of this study is that in comparison with single-dose of TXA, multiple-dose of TXA was associated with significantly shortened R times and marginally aggravated hypercoagulable state, but this prothrombotic state does not provoke thrombosis, especially when combined with appropriate anticoagulant therapy.

### Comparison with other studies

Although TXA has been widely used in arthroplasty and is considered to have a good safety profile, challenges have remained ever since. TEG as a whole blood measure of coagulation has become accessible and viable over the last few decades [27]. A previous study explored the effects of TXA on TEG parameters in patients receiving cardiac surgery, which found that the use of TXA was associated with a shortening of the R time and no significant effect on K, α-angle, or MA values [28]. Another study assessed the effects of TXA on hematological, hemostatic, and TEG analytes in healthy dogs, which also found a significant decrease in *R* value [29]. Our study compared multiple-dose versus single-dose of TXA in TJA, and found consistent results with previous research. However, the potential mechanisms have not been fully elaborated.

### Possible mechanisms

TXA is synthetic derivative of the amino acid lysine, which acts as a potent competitive inhibitor of plasminogen activation by reducing the binding of plasminogen to fibrin, and inhibiting active fibrinolytic enzyme plasmin formation by tissue plasminogen activator [30]. The decreased conversion of plasminogen to plasmin results in inhibited breakdown of fibrinogen to fibrin, reduced enzymatic degradation of fibrin blood clots, and finally reduces the blood loss [31].

Apart from the role in fibrinolysis, plasmin also interacts with many coagulation factors; these complex interactions and feedback mechanisms would induce procoagulant activity [32]. On the one hand, multicoagulation factors, especially coagulation factors FV and FVIII, are initially activated by plasmin and followed by rapid inactivation [33]. On the other hand, plasmin might also directly promote the generation of thrombin by breakdown of the tissue factor protein inhibitor, a major inhibitor of tissue factor-mediated coagulation [34]. Overall, plasmin would increase thrombin production, and the inhibition of plasmin generation by TXA would reduce thrombin generation. But the initial activation might have generated enough thrombin to produce a significant procoagulant effect [34]. The shortened R time may associate with the reduced inactivation of coagulation factors by plasmin.

On top of that, TXA could improve the platelet function in certain circumstances, especially for patients with impaired platelet function [35–38]. However, our participants exhibited normal platelet function, and we did not find platelet hypercoagulability in the multiple-dose cohort when comparing with the single-dose cohort, even platelet count has been substantially conserved. Platelet would be activated by plasmin via different mechanisms, such as stimulating the complement cascade, mediating platelet degranulation, inducing arachidonic acid cascade, and activating the protease-activating receptor-4 [34]. Multiple-dose of TXA might reduce plasmin, decreases the multifactorial activation of platelets, balances the increased platelet count, and consequently inducing similar MA values with single-dose cohort.

### Implications for clinical practice

Given the universal implementation of thromboprophylaxis and the rarity of DVT in patients undergoing arthroplasty, it would be extraordinarily difficult to obtain a statistically powered sample size to reach a firm conclusion from an evidence-based approach. We thus applied TEG, a more sensitive method to detect the potential consumptive coagulopathy and assess the safety of TXA. Our study demonstrated that multiple-dose of TXA was associated with significantly shortened R times and marginally aggravated hypercoagulable state. Our findings suggest that TXA appears to induce procoagulant activity in coagulation factors and raises the risk of DVT. This situation would be worse when compounding with other prothrombotic factors, and routine use of pharmacological prophylaxis might mitigate the hypercoagulability and alleviate the risk of thromboembolism. Therefore, potential benefits and possible risks should remain a trade-off when considering increasing the dosage or frequency of TXA.

Additionally, the difference between THA and TKA should also be noted. In this study, we included both hip fracture and non-hip fracture patients. Patients with hip fractures represent a high-risk group; the hip fracture has a direct impact on coagulation system and significantly changed the pre-operative coagulation screening tests results [39–41]. Therefore, the population who received THA tends to be more heterogeneous than TKA, which leads to greater variance of the tests results than patients who received TKA.

### Call for future studies

Considering the contemporary challenges and unresolved issues, future explorations that aim to redefine the safety of TXA are warranted. First, prospective randomized controlled trials or cohort studies are still needed to confirm our findings and to elucidate the underlying mechanisms of TXA. As a competitive inhibitor of plasminogen activation, little is known about the minimal effective dose or most effective and safe dose of TXA for patients undergoing arthroplasty. Therefore, the mechanisms of TXA are desperately needed to guide the application of TXA. Second, the balance of hemostasis and anticoagulation is an international state-of-the-art in arthroplasty. Our study found a hypercoagulable state after multiple-dose of TXA, and previous animal research also discovered that TXA dose-dependently increased thrombus formation and thrombus weight [42], which highlighted the essential of thromboprophylaxis especially pharmacological prophylaxis. Given that the dosages and administration schedules of TXA varied widely among studies, and there is an ascending trend in the dosage and frequency of TXA in clinical practice, guidance on how to adjust the pharmacological prophylaxis accordingly to balance the risk of bleeding and thrombosis is desperately needed.

### Strengths and limitations

To the best of our knowledge, this is the first study that applied TEG to evaluate the safety of TXA in arthroplasty, which assessed the safety of TXA with a new approach.

Our study has several limitations. First, this study is limited by the fact that it is a retrospective review of two different cohorts over two separate time periods. Thus, our results could be affected by the heterogeneity of participants and clinical practice, although we have applied strict inclusion/exclusion criteria to control the influential factors. Second, we did not have favorable follow-up data especially ultrasonography examination results, since many patients chose to follow-up at local hospitals due to inconvenient transportation in our mountain areas.

## Conclusions

Compared with single-dose of TXA, multiple-dose of TXA was associated with significantly shortened R times and slightly aggravated hypercoagulable state, but this prothrombotic state does not provoke thrombosis, especially when combined with appropriate anticoagulant therapy. Therefore, multiple-dose of TXA remains safe and could be recommended for clinical practice. Potential benefits and possible risks should be a trade-off when considering increasing the dosage and frequency of TXA on the present basis.

## Abbreviations

APTT: Activated partial thromboplastin time
ASA: American statistical association
AVN: Avascular necrosis
BMI: Body mass index
CI: Coagulation index
DDH: Developmental dysplasia of the hip
DVT: Deep vein thrombosis
FAI: Femoroacetabular impingement
Fbg: Fibrinogen
FNF: Femoral neck fracture
GA: Gout arthritis
Hb: Hemoglobin
ITF: Intertrochanteric fracture
IV: Intravenous
K: Kinetics
MA: Maximum amplitude
OA: Osteoarthritis
PE: Pulmonary embolism
POD: Post-operative day
Pre: Pre-operatively
PT: Prothrombin time
R: Reaction time
RA: Rheumatoid arthritis
ROTEM: Thromboelastometry
STROCSS: Strengthening the Reporting Of Cohort Studies in Surgery
TEG: Thrombelastography
THA: Total hip arthroplasty
TJA: Total joint arthroplasty
TKA: Total knee arthroplasty
TT: Thromboplastin time
TXA: Tranexamic acid
VTE: Venous thromboembolic
